# Deswelling and deformation of microgels in concentrated packings

**DOI:** 10.1038/s41598-017-10788-y

**Published:** 2017-08-31

**Authors:** I. Bouhid de Aguiar, T. van de Laar, M. Meireles, A. Bouchoux, J. Sprakel, K. Schroën

**Affiliations:** 1 Laboratory of Food Process Engineering, Wageningen University & Research, Wageningen, The Netherlands; 2 Laboratoire de Génie Chimique, Université de Toulouse, CNRS, INPT, UPS, Toulouse, France; 3 Physical chemistry and Soft matter group, Wageningen University & Research, Wageningen, The Netherlands; 4 Laboratoire d’Ingénierie des Systèmes Biologiques et des Procédés, Université de Toulouse, CNRS, INRA, INSA, Toulouse, France

## Abstract

Increasing the particle density of a suspension of microgel colloids above the point of random-close packing, must involve deformations of the particle to accommodate the increase in volume fraction. By contrast to the isotropic osmotic deswelling of soft particles, the particle-particle contacts give rise to a non-homogeneous pressure, raising the question if these deformations occur through homogeneous deswelling or by the formation of facets. Here we aim to answer this question through a combination of imaging of individual microgels in dense packings and a simple model to describe the balance between shape versus volume changes. We find a transition from shape changes at low pressures to volume changes at high pressures, which can be explained qualitatively with our model. Whereas contact mechanics govern at low pressures giving rise to facets, osmotic effects govern at higher pressures, which leads to a more homogeneous deswelling. Our results show that both types of deformation play a large role in highly concentrated microgel suspensions and thus must be taken into account to arrive at an accurate description of the structure, dynamics and mechanics of concentrated suspensions of soft spheres.

## Introduction

Microgels are colloidal particles made from a solvent-swollen crosslinked polymer network^1, 2^, whose softness can be tuned with the crosslink density^3^. These microgels are commonly used as a well-defined experimental model system to explore the phase behaviour, dynamics and mechanics of soft particle suspensions^4, 5^. Their softness, which entails both a low resistance to shape and to volume changes, has a large effect on the properties of dense suspensions of these particles. For example, they can be compressed to packing density in excess of random close packing and their increase in viscosity as they approach the liquid-solid boundary shows significant deviations from the behaviour of hard spheres^6, 7^. Moreover, microgels exhibit a rich phase behaviour^8–10^, which can be tailored by their degree of crosslinking^11^, the presence of charges^12^ or inherent network inhomogeneities^13^.

To achieve effective packing densities of well above the random close packing limit for hard spheres, the reduction in available volume must be accommodated by either shape or volume changes in the constituent particles. This can occur either by forming facets at the contact points with the surrounding particles^8, 14, 15^ and by the expulsion of solvent from the particle, leading to homogeneous deswelling and volume reduction^16, 17^. Recent work has highlighted how the latter can have pronounced effects on the interpretation of experiments on microgels, since osmotic deswelling can lead to substantial deviations between the apparent and real particle volume fraction^18^.

It is most likely that facetting and homogeneous deswelling are relevant to some extent; however, this remains relatively unexplored. Recent contrast-variation scattering experiments have shed light on this complexity for the first time, showing an interplay of deformations, deswelling and even interpenetration of surface-dangling chains as the particle concentration is varied^19^. Yet, our quantitative understanding of particle deswelling and deformation remains incomplete.

The isotropic compression of individual microgels subjected to a homogeneous osmotic force has been studied in detail previously. For example, microgels suspended in solutions of a polymeric osmolyte, such as dextran which is excluded from the microgel network, exhibit a homogeneous osmotic deswelling consistent with polymer swelling theory^6^, from which the bulk modulus *K* of the individual microgels could be determined. Squeezing a single microgel between two sapphire plates, yielding two discrete contact points, has shown that this is a controlled way of probing deformations of single particles, but the possibility of an interplay between shape and volume changes was not discussed^17^. In this last case, the microgel is under non-homogeneous pressure. This implies that contact deformations cannot be ignored as it is also the case for shape or volume changes, dictated by the Poisson ratio, which is typically between 0.4–0.45 for hydrogel particles^20^. Of course, the same argument holds for particles with more than two contact points, as would be the case in a dense packing of particles, contacting multiple neighbours. The fact that both effects contribute to microgel shape and size in non-homogeneous pressure fields is illustrated by the capillary micromechanics work of Guo and Wyss^5^ where individual soft particles are brought into a tapered confinement, which induced both shape and size changes, that can be quantified accurately, for example to derive the full linear mechanics of single particles.

So while it is clear that the mechanical response of compressible and deformable microgels to complex pressure fields involves both shape and size changes, these effects remain to be explored in dense packings of many microgels in close contact. Understanding these effects is an important step towards a more comprehensive description of the combined effects of single-particle mechanics and osmotic equilibrium on the properties of concentrated suspensions of soft particles.

In this paper we explore the deformation and deswelling mechanisms of microgels in compressed microgel packings and provide a framework to understand their behaviour. We osmotically stress mixtures of fluorescent and non-fluorescent microgels and image the shape and size of single microgels with high resolution using confocal microscopy and quantitative analysis algorithms. We find that the ratio of shape to volume changes, evolves non-monotonically with applied pressure; at low pressures shape changes are pronounced, in the form of facets, while at larger pressure the facets disappear and the microgels assume a spherical shape by deswelling homogeneously. We qualitatively explain these results using a simple mechanical model, which combines the osmotic pressure of the gel network with contact mechanics.

## Results and Discussion

We study microgels made from poly(acrylamide) (pAAm) prepared by emulsion templating. The particles are crosslinked with 1%wt of crosslinker with respect to the total monomer content, resulting in reasonably soft microgels. Here we aim to prepare microgels with sizes larger than 10 *μ*m such that their shape and size can be carefully deduced from confocal fluorescence microscopy experiments. Although our microgels are large enough to be imaged by brightfield microscopy, it remains challenging to obtain the entire shape and size due to significant artefacts that arise due to the high particle concentration. Rather, we choose to use confocal microscopy to vizualize a few labelled microgels in a large excess of undyed particles. This enables us to resolve the microgel shape and size with high resolution and without being hindered by the high particle density. To concentrate the microgel suspensions to a well-defined macroscopic osmotic pressure, we stress the suspensions by placing them in a dialysis membrane and equilibrating them against poly(ethylene glycol) (PEG) as an osmolyte, which leads to a homogeneous compression of the suspension to osmotic pressure differences between 10^3^–10^6^ Pa.

**Figure 1:**
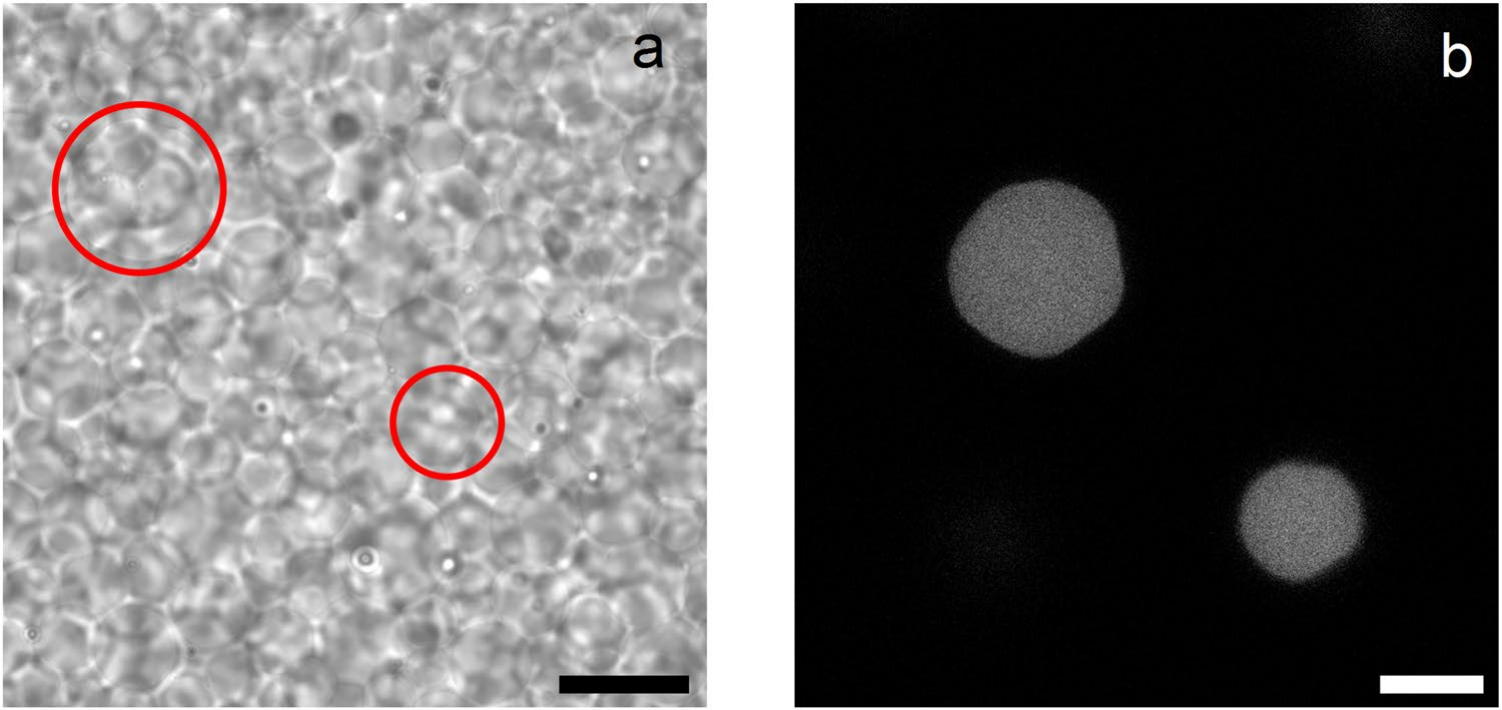
Compressed microgel packing composed of a mixture of fluorescent and non-fluorescent microgels. (**a**) Bright field image. Scale bar denotes 20 *μ*m. (**b**) CLSM image. Scale bar denotes 10 *μ*m. The images were made at the same region of the sample. Red circles in the bright field image indicate the position of the fluorescent particles but are not to scale.

If all the microgels are fluorescently labelled, observing the boundaries of a single particle at its contacts with neighbours becomes highly inaccurate. To end this, we use a mixture of fluorescent and non-fluorescent microgels in our experiments. This allows us to accurately observe a single fluorescent microgel that is surrounded by non-fluorescent microgels. While identification of single particles is difficult in bright-field microscopy images (Fig. 1a) where all particles provide contrast, well-defined images of single fluorescent particles can be made using confocal microscopy, as shown in Fig. 1b.

**Figure 2:**
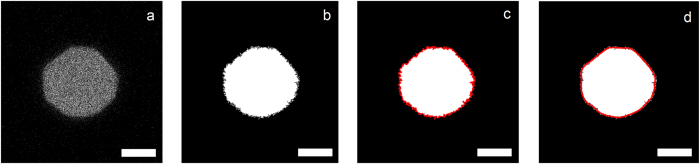
Steps during image treatment, (**a**) shows the raw microscopy data, (**b**) shows the binary version of this image, generated to most closely preserve the microgel shape, (**c**) shows a simple edge trace of this binary image, resulting in many artifacts, (**d**) shows the much smoother tracing after fitting this boundary with a Savitzky-Golay filter, which results in a smooth boundary while preserving the overall microgel shape. Scale bars denote 5 *μ*m.

To quantify changes in size and shape of the individual microgels in the packings, we image at least twenty separate microgels in three-dimensions using confocal microscopy for each compression pressure. From these images, we can calculate the microgel volume and shape. In order to calculate an accurate perimeter and area for each slice in the three-dimensional image stacks, we first convert our images (Fig. 2a) to binary black and white (Fig. 2b). During this thresholding, pixelation at the background-particle edge results in jagged edges in an edge-detection algorithm (Fig. 2c). Such roughness on the perceived perimeter would overestimate the particle contour. This discretization effect can be minimized by recording high-resolution confocal images, but some boundary effects remain. To solve this issue we first trace this perimeter using a Savitsky-Golay (SG) filter^21^ to smooth the boundary (Fig. 2d). From these smoothed traces we then reconstruct the image, which allows us to calculate the perimeter and area much more accurately, while still having access to the overall microgel shape without blunting due to the filtering.

**Figure 3:**
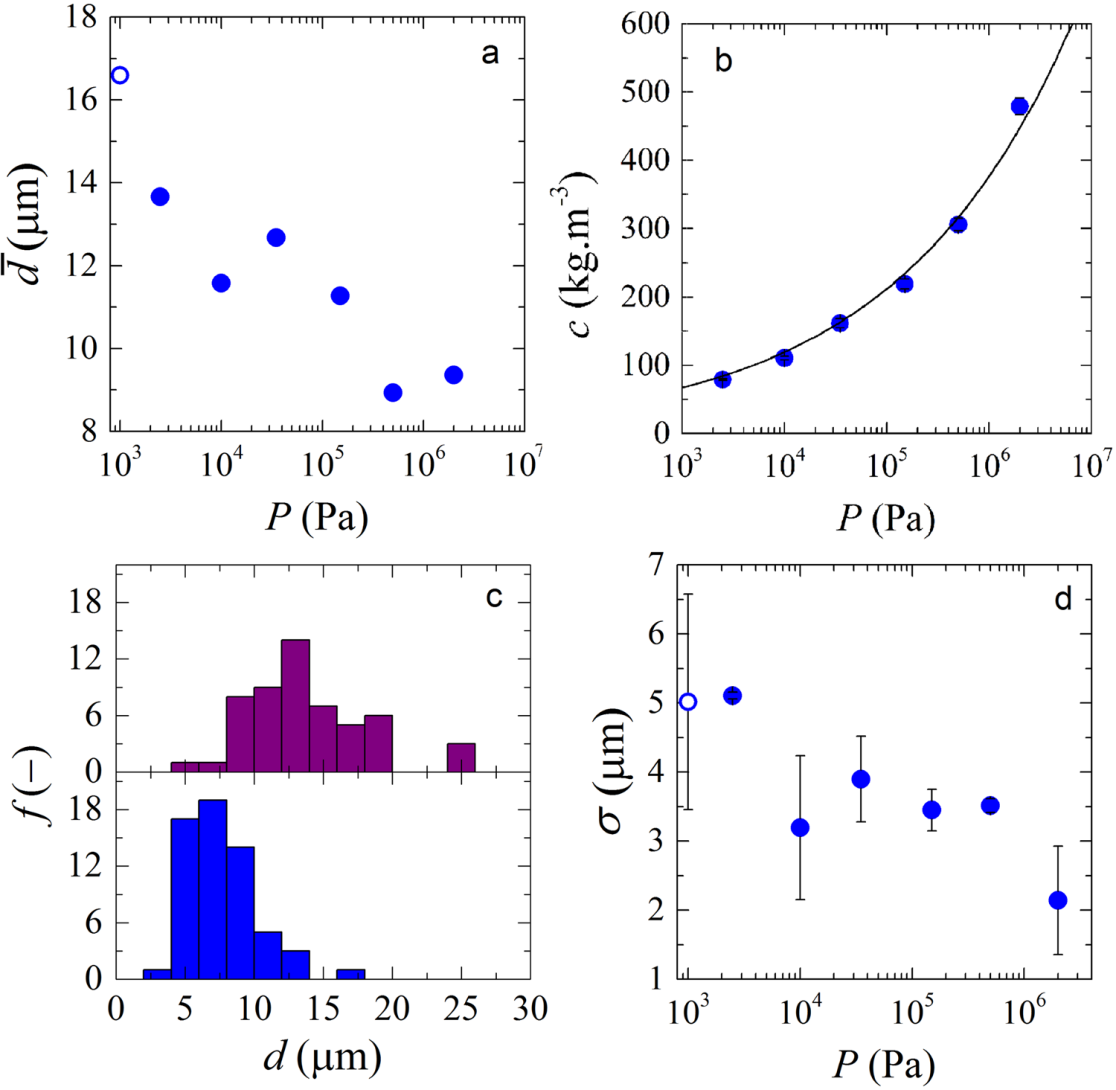
(**a**) Average microgel diameter ($\overline{d}$) as a function of compression pressure, (**b**) microgel packing polymer concentrations *c* as a function of pressure *P*. Solid line describes the $P\propto {c}^{4}$ scaling. (**c**) size distributions of microgel diameter (*d*) for two compression pressures (2.5 kPa - top and 2 MPa - bottom), which shows a narrowing of the size distribution at increased compression and (**d**) width of fitted Gaussian functions (σ) for each size distribution as a function of compression pressure. Open symbols are values obtained at zero pressure (*P*).

We first probe the changes in particle volume, to evaluate their osmotic deswelling in response to the contact pressure of neighbouring particles. We deduce the particle volume from the equivalent sphere diameter $\overline{d}$ fitted to the three-dimensional image stacks of thresholded and filtered images of single particles (more details in the Material and Methods section). As expected, we observe that the average size of the microgels decreases with increasing compression pressure, as a result of solvent expulsion by the microgels (Fig. 3a). The average values were obtained by averaging over multiple particles in a polydisperse population; nevertheless, we see a clear monotonic trend of deswelling with increasing pressure *P*, consistent with previous reports^17, 22^.

For each compression pressure *P*, we also determine the polymer concentration in the compressed microgel packings by dehydrating the particle pastes and measuring the dry weight (Fig. 3b). We find that the osmotic pressure increases steeply with increasing polymer concentration. The data is well described by a scaling $P\propto {c}^{4}$, which is significantly higher than the scaling prediction for the osmotic pressure of a semi-dilute polymer solution within the blob model of $P\propto {c}^{\mathrm{9/4}}$ ^23^. We attribute this to the additional contribution of network elasticity to the osmotic pressure, as described by the Flory-Rehner theory^24^, where the rise in osmotic pressure with concentration, in particular close to the equilibrium swelling state of the particles, is much steeper than that of a simple solution of linear chains^25^.

From our experimental observations, we do not only have access to the average particle size as a function of compression, but also the size distribution. Interestingly, we see how the size distribution shifts as the compression increases (Fig. 3c). This is likely due to the fact that larger microgels will be more compressed in the packings, whereas small particles can reside in interstitial spaces and thus experience smaller contact pressures on average. As a consequence, larger particles will deswell more than smaller ones, thus narrowing the size distribution of the sample. To quantify the change in size distribution, we measure the width of the particle size distributions *σ* as the full width at half maximum (FWHM) by fitting the experimental data to a normal distribution at all compression pressures. Indeed, the width of the distribution decreases with increasing pressure (Fig. 3d). We also plotted (results not shown) the ratio between the gaussian width and the mean with varying pressure and obtained the same decreasing trend. The fact that we obtained the same trend shows that the decrease in size of the particles is not the reason for the narrowing of the size distribution. This observation of a narrowing particle size distribution is consistent with earlier reports of a co-crystallisation of large microgels in a bath of smaller particles as the pressure increased, leading to shrinkage of the larger particles to fit into the microgel lattice^26^.

Clearly, increasing the particle density leads to pronounced osmotic deswelling of the particles. However, visual inspection of the confocal microscopy images shows also how distinct facets develop at the particle-particle contact points (Fig. 2). While most previous studies have studied in-depth the volume changes associated with osmotic compression of microgel packings, these shape changes have received much less attention so far, but may be crucial to understand the rheology and dynamics of microgel pastes.

To evaluate the extent of shape changes, we determine to what extent the particle shape deviates from a perfect sphere. Due to the preparation templated in emulsion droplets, the rest shape of the microgels is a near-perfect sphere. We define the sphericity, extracted from our two-dimensional confocal images as:

1 $${\rm{\Psi }}=\frac{2\sqrt{\pi {N}_{a}}}{{N}_{circ}}$$

where *N*_*a*_ is the number of pixels in the area enclosed by the SG filtered boundary (red line in Fig. 2d) and *N*_*circ*_ is the number of pixels along the boundary contour. For a perfect circle ${\rm{\Psi }}\equiv 1$, while any asphericity, e.g. due to facetting, will result in ${\rm{\Psi }} < 1$.

**Figure 4:**
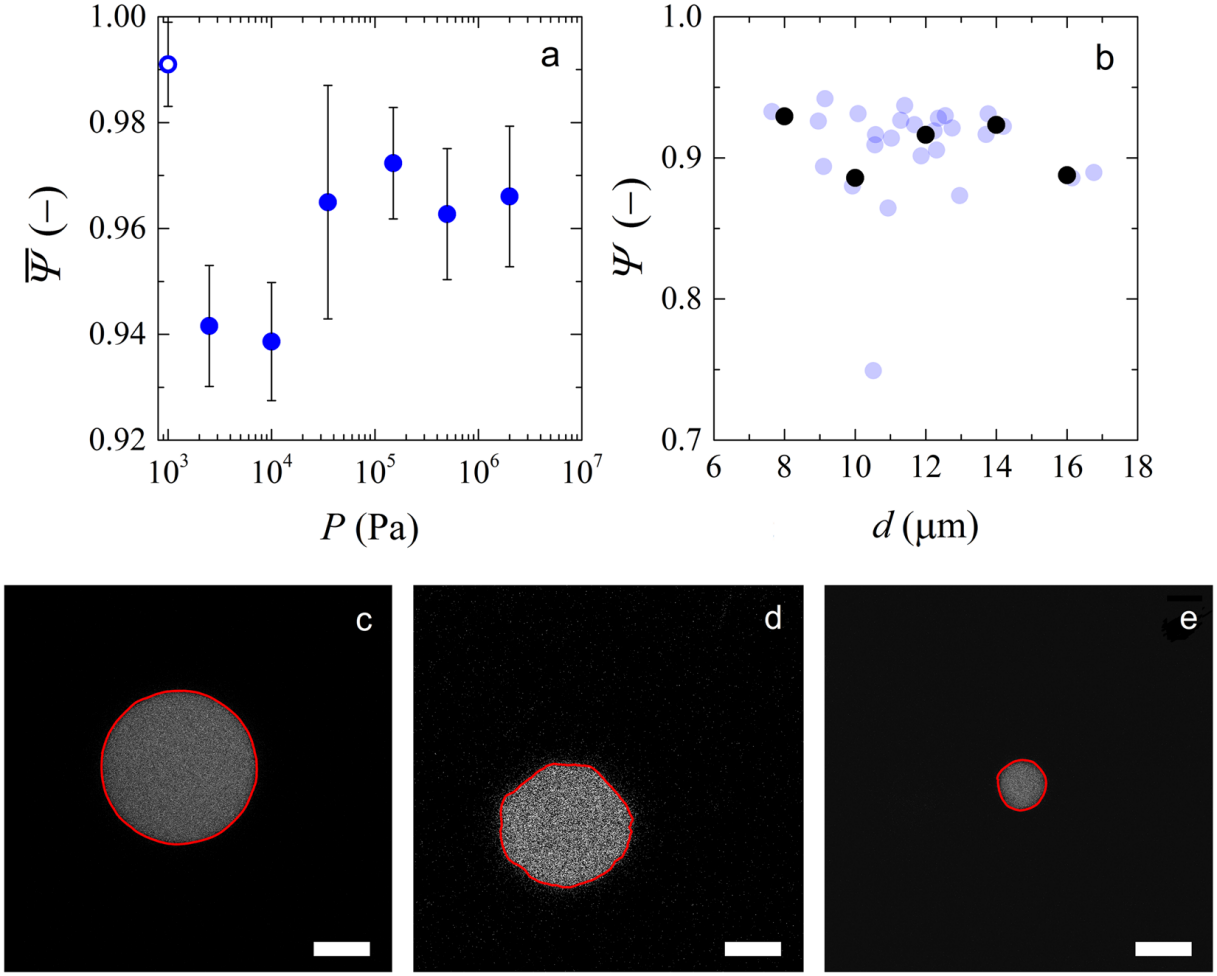
(**a**) Average sphericity ($\overline{{\rm{\Psi }}}$) as a function of compression pressure. The open symbol is a value obtained at zero pressure *P* = 0. (**b**) Microgel sphericity (Ψ) as a function of microgel diameter (*d*) for a single compression pressure (10 kPa). Black circles are binned data. Bin width is 2 and the values within each bin were averaged. Confocal images of a single microgel at (**c**) zero pressure, (**d**) at *P* = 10 kPa and (**e**) 2 MPa. Red outlines correspond to the edges of the particle after image analysis. Scale bars denote 5 *μ*m.

Samples at zero pressure *P* = 0, exhibit an almost perfect spherical geometry with ${\rm{\Psi }}\approx 0.99\pm 0.1$ (Fig. 4c); the small deviation from Ψ = 1 is caused by the inevitable discretization of the images at the scale of a pixel, which cannot be completely circumvented by the SG filtering of the particle contour.

At finite pressure, the sphericity, averaged over at least twenty particles at each pressure, initially decreases. This indicates that the microgels become deformed by the formation of facets at contact points with neighbouring particles (Fig. 4d). As the compression pressures *P* increase, the average sphericity of the microgels increases again, which indicates that the particles regain their spherical shape (Fig. 4e). For comparison, we evaluated images containing polygons and obtained sphericity values for an hexagon and a square of 0.97 and 0.91, respectively. We also evaluated images of spheres of different sizes to rule out the effect of the amount of pixels constituting the sphere in the sphericity values. We found that for the size of images used (1000 × 1000 pixels), or the size of the sphere did not interfere in the sphericity results. Finally, to investigate whether the size polydispersity of the microgels has an influence on their degree of deformation, we plot the sphericity Ψ as a function of the diameter of the microgels *d* (Fig. 4b) for a certain compression pressure (10 kPa). We find no statistically significant trend, indicating that there is no significant effect of size polydispersity on the particle deformations, within the statistical noise of our experiment.

Our experimental results show that both faceting and deswelling happen, depending on the applied pressure, in a distinctly non-monotonic way. As the pressure increases, facets first become more pronounced, until they start to become less noticeable and the particle appears to homogeneously deswell to a (smaller) spherical configuration. This counterintuitive observation triggers the question if these are equilibrium effects, or whether non-equilibrium aspects may be important. First, we note that the samples are equilibrated for 14 days at a given osmotic pressure. The timescales for poroelastic relaxation, i.e. the solvent flow within the porous polymer particles required to achieve shape and size changes, occurs on much smaller time scales, and are thus not likely to contribute. Moreover, experiments conducted at different times give identical results, suggesting time-dependencies not to be of significant influence.

To confirm that the change from facetting at low pressure to osmotic deswelling at higher pressures is an equilibrium effect, we derive a simple equilibrium model that is capable of reproducing the observed behaviour by balancing contact mechanics versus osmotic effects upon creating particle-particle contacts.

Since the experimental microgel particles of polyacrylamide are under good solvency conditions, we derive an extension on the classical description of Flory and Rehner^27, 28^, which assumes ideal chains between nodes that are marginally stretched, to account for large chain extensions. The osmotic pressure within a microgel particle results from two opposing terms. The first is a mixing term, describing the mixing entropy and the enthalpy of solvent-monomer interactions, which promotes swelling. Within the mean-field Flory-Rehner approach this can be written as:

2 $${{\rm{\Pi }}}_{mix}=\frac{{k}_{B}T}{{a}^{3}}(-\phi -\,\mathrm{ln}\,\mathrm{(1}-\phi )-\chi {\phi }^{2})$$

where k_*B*_ *T* is the thermal energy, *a* the size of a statistical chain segment, *χ* the Flory interaction parameter and φ the monomer volume fraction, which is the main control parameter. This term is always positive and as such promotes the uptake of solvent within the microgel particle.

The mixing pressure is balanced by the elasticity of the chain segments between crosslinks. Swelling stretches the chains between crosslinks which reduces their conformational entropy. Traditionally, within the Flory-Rehner description, this entropic elasticity is estimated within the Gaussian approximation, which assumes that chains obey a Hookean force law. However, this is only valid when the distance between two crosslinks *ξ* is close to the relaxed dimension of the chains *R*_*g*_. For strongly swollen microgels however, chain extension between nodes may be strong, where large deviations from Hookean behaviour may be expected.

To capture this limit as well, we use the freely-jointed chain (FJC) model, which describes the elastic force *F* on a polymer chain extended to length *ξ* as:

3 $$F=\frac{\beta {k}_{B}T}{a}$$

in which *β* is the inverse Langevin function, that can be expanded as:

4 $$\beta =3(\frac{\xi }{{N}_{x}a})+\frac{9}{5}{(\frac{\xi }{{N}_{x}a})}^{3}+\frac{297}{175}{(\frac{\xi }{{N}_{x}a})}^{5}+\ldots $$

where *N*_*x*_ is the number of statistical segments between crosslinks. In the limit of small chain extensions this returns to the Gaussian result for which the Hookean spring constant $k={k}_{B}T/{N}_{x}{a}^{2}$ is valid. Particle swelling, thereby increasing *ξ*, leads to an effective elastic pressure to counteract swelling:

5 $${{\rm{\Pi }}}_{el}=\frac{F}{{\xi }^{2}}=\frac{\beta {k}_{B}T}{a{\xi }^{2}}=\frac{{k}_{B}T}{a}(\frac{3}{\xi {N}_{x}a}+\frac{9\xi }{\mathrm{5(}{N}_{x}a{)}^{3}}+\frac{297{\xi }^{3}}{\mathrm{175(}{N}_{x}a{)}^{5}}+\ldots )$$

where the monomer volume fraction is related to the characteristic mesh size as $\phi ={N}_{x}{a}^{3}/{\xi }^{3}$.

As the microgel is dissolved in a solvent, thermodynamic equilibrium requires the balancing of the pressure within the particle ${{\rm{\Pi }}}_{in}$, by swelling or deswelling, with the external osmotic pressure ${{\rm{\Pi }}}_{ex}$:

6 $${{\rm{\Pi }}}_{in}={{\rm{\Pi }}}_{mix}-{{\rm{\Pi }}}_{el}={{\rm{\Pi }}}_{ex}$$

We define the relaxed reference state of the microgel as ${{\rm{\Pi }}}_{in}={{\rm{\Pi }}}_{ex}=0$, where the polymer volume fraction within the particles $\phi ={\phi }_{0}$ The bulk modulus *K* is defined as:

7 $$K=\phi \frac{{{\rm{d}}{\rm{\Pi }}}_{in}}{{\rm{d}}\phi }$$

The resistance of the same particle against shape changing deformations, typically by the formation of facets at the contacts of a particle with its neighbours, can be defined by its Young’s modulus *E*, defined as:

8 $$E=3K(1-2\nu )$$

with *ν* the Poisson’s ratio of the hydrogel particles. More comprehensive micromechanical mean-field approaches to explore the effect of particle elasticity and compressibility on microgel glasses both at rest and under shear have been reported recently^18, 29^.

**Figure 5:**
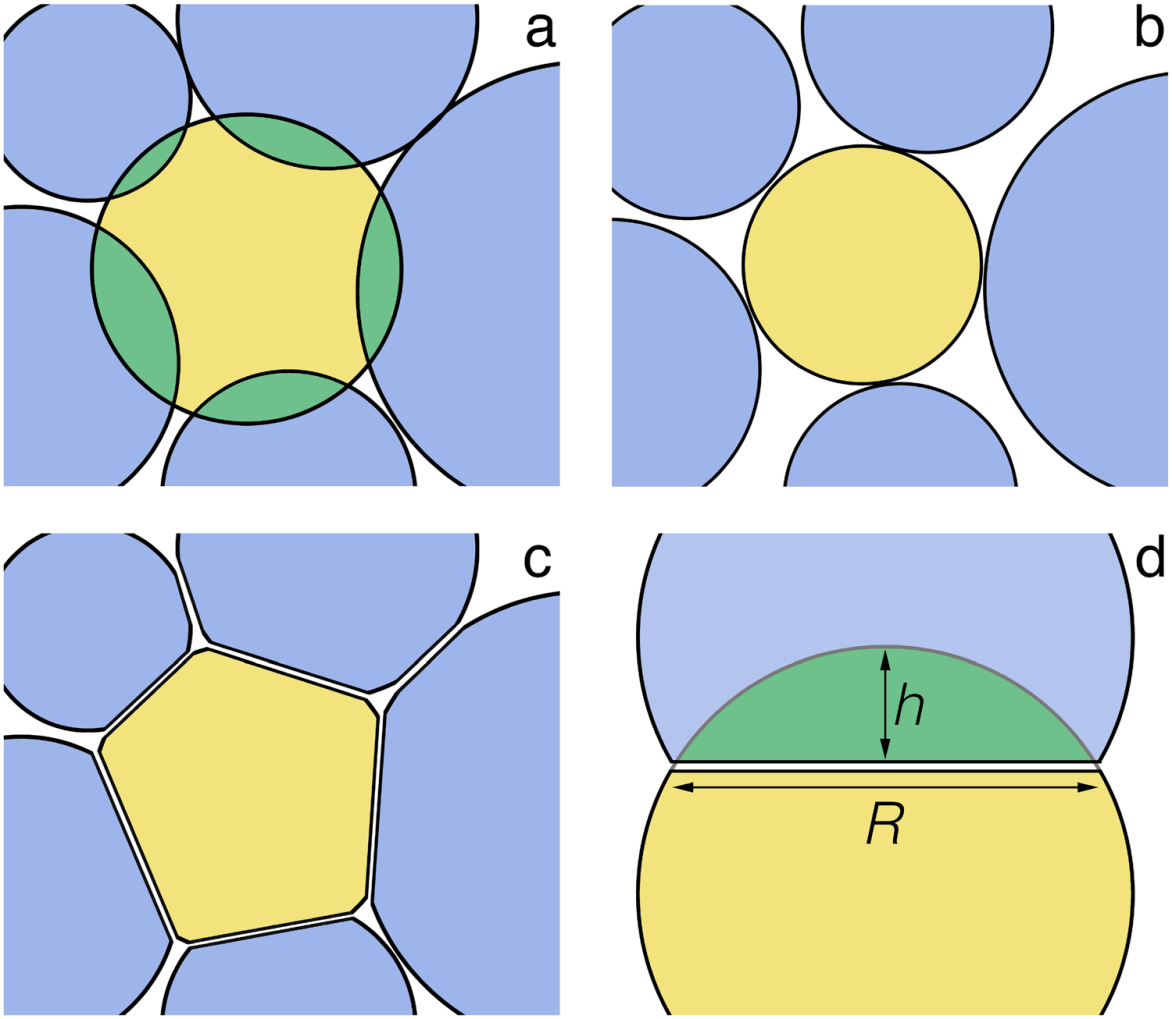
Osmotic compression of a microgel suspension leads to unphysical overlap between neighbours (**a**), which is resolved either by deswelling the particles (**b**) or through particle deformation in the form of contact facets (**c**). In the calculation of the energy of deformation by facet formation *U*_*f*_ we use a Hertzian contact model in which the deformation is approximated by the overlap with penetration depth *h* leading to facets of size *R* (**d**).

Upon increasing the pressure of a microgel suspension, physical overlap between the particles (Fig. 5a) must be avoided, either by shrinking or the formation of facets (Fig. 5b,c). To evaluate the extent of both of these modes of response to compression we consider the work of deformation due to faceting *W*_*d*_ and the work of shrinkage *W*_*s*_; both of these represent the reversible (thermodynamic) work performed on a central particle at a given number of particles, total volume of the system and temperature.

The overlap *h* between two neighbouring particles is defined in Fig. 5d, which can be resolved by a linear combination of contributions due to deformation *h*_*d*_ and shrinking *h*_*s*_: $h={h}_{s}+{h}_{d}$. The fraction of the response attributed to faceting-type deformations can thus be formulated as: ${\alpha }_{d}=\frac{{h}_{d}}{{h}_{d}+{h}_{s}}$, and the fraction contributed to shrinking as $\mathrm{(1}-{\alpha }_{d})$.

The work of shrinkage is given by:

9 $${W}_{s}={{\rm{\Pi }}}_{in}{\rm{\Delta }}V=\frac{4\pi }{3}{{\rm{\Pi }}}_{in}({{R}_{0}}^{3}-{({R}_{0}-{h}_{s})}^{3})$$

with *R*_0_ the radius of the microgel in dilute conditions, where ${{\rm{\Pi }}}_{in}=0$.

The work of deformation by forming facets is gauged by using the Hertzian model for the elastic contact between two spheres of equal size *R*_0_. The force required to form an indentation of depth *h* is given by:

10 $$F=\frac{4}{3}E{{R}_{0}}^{\mathrm{1/2}}{h}^{\mathrm{3/2}}$$

such that the work required to perform a deformation of depth *h*_*d*_ between two spheres becomes:

11 $${W}_{d}={\int }_{0}^{{h}_{d}}F(h)dh=\frac{8}{15}E{{R}_{0}}^{\mathrm{1/2}}{{h}_{d}}^{\mathrm{5/2}}$$

Since each microgel particles has *Z* neighbours, the total work associated with deformations becomes:

12 $${W}_{d}=\frac{8}{15}E{{R}_{0}}^{\mathrm{1/2}}{{h}_{d}}^{\mathrm{5/2}}Z$$

The total mechanical work can now be defined as:

13 $$W={\alpha }_{d}{W}_{d}+(1-{\alpha }_{d}){W}_{s}$$

These two contributions need to be balanced to minimize the overall mechanical work. Thus to find the relative amounts of deformation and shrinkage, we must solve:

14 $$\frac{dW}{d\alpha }=0$$

This allows us to evaluate for each pressure, given our expressions for the microgel elasticity and the mechanical work upon compression to what extent a particle will deform and shrink, as expressed by the parameter *α*_*d*_. If *α*_*d*_ ≈ 1 the particle will solely deform and shrinkage is negligible; by contrast if *α*_*d*_ ≈ 0, only isotropic shrinkage occurs while the particles maintain their spherical shape.

To compute the elastic properties of the microgels, we need to choose values for the three independent parameters which govern the microgel properties: i) *N*: the number of statistical segments between crosslinks, for which we use *N* = 250 (note that the behaviour we observe is robust to the choice of the crosslinking density and is mostly sensitive to the Poisson ratio of the hydrogel). ii) *a*: the size of a statistical unit, for polyacrylamide microgels as the experimental example, we use the Kuhn length of polyacrylamide as $a\sim 0.4$ nm^30^, iii) *χ*: the Flory interaction parameter describing the interactions between polymer and solvent, for polyacrylamide in water at room temperature $\chi =0.48$ ^31^. For the equilibrium particle size at rest *R*_0_ we take 5 *μ*m as also used in our experiments. Even though the coordination number is known to vary with particle concentration^32^, for the sake of simplicity we assume *Z* = 12, corresponding to the close-packed limit for monodisperse spheres; also here we find that the results are robust to the choice of *Z*.

**Figure 6:**
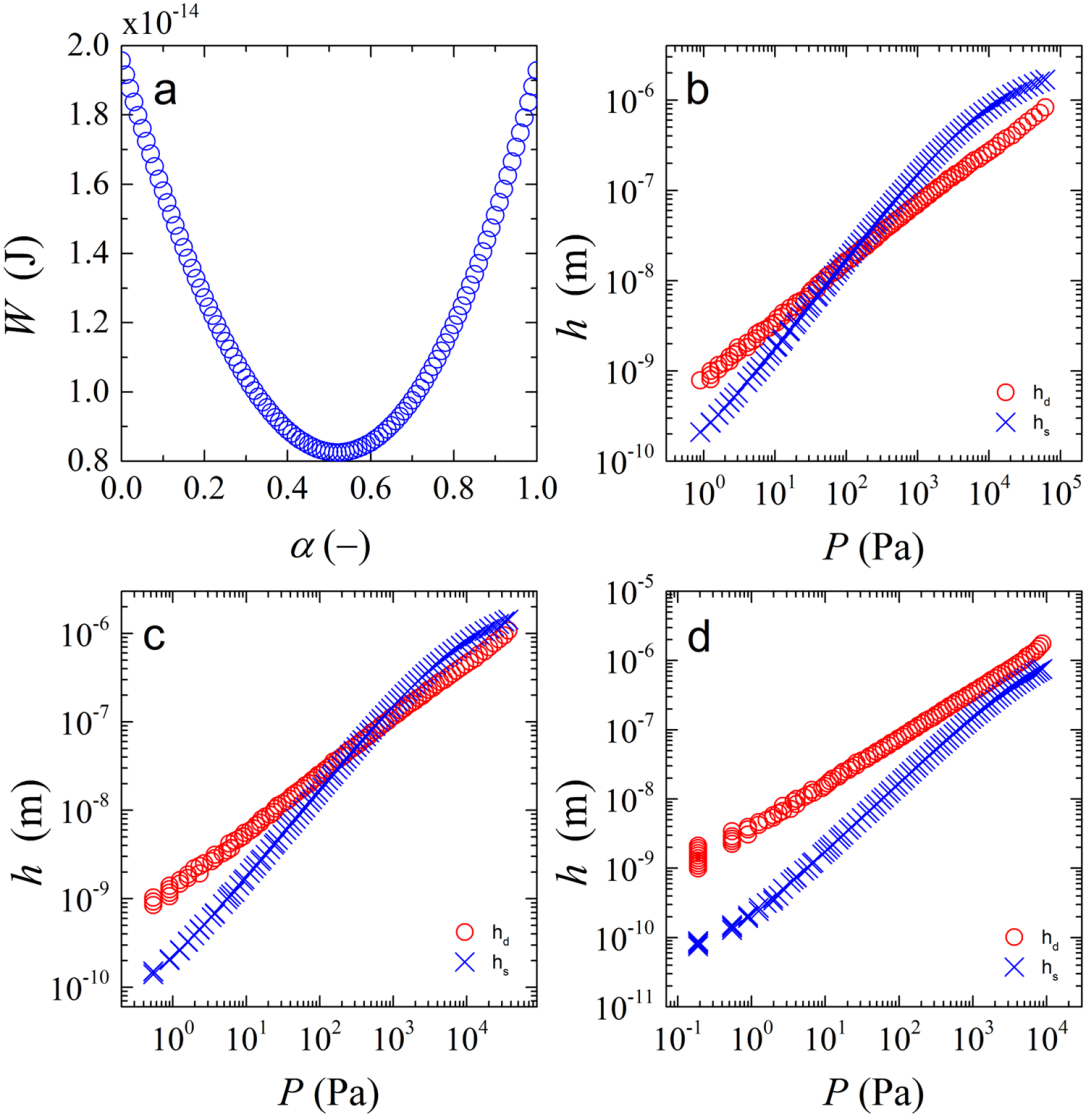
(**a**) Mechanical work *W* as a function of the fraction of the overlap that is mitigated through deformation $\alpha ={h}_{d}/({h}_{s}+{h}_{d})$, showing a clear minimum where $dW/d\alpha =0$, signalling the equilibrium condition (for: $\nu =0.40$ and ${h}_{s}+{h}_{d}=100$ nm), (**b**–**d**) Contributions of shrinkage *h*_*s*_ and deformation *h*_*d*_ to the total particle response as a function of the total pressure P for three different values of the Poisson’s ratio *v* = 0.3 (**b**), 0.4 (**c**) and 0.48 (**d**).

Indeed we see that the mechanical work *W* has a minimum when plotted as a function of *α*, the fraction of the overlap *h* mitigated by means of faceting (Fig. 6a). By finding this minimum, we can now assess the relative contributions of shrinkage and deformation as a function of the applied pressure.

Interestingly, the experimental behaviour is qualitatively reproduced within our approximate theory. When we plot the amount of overlap *h* that is mitigated by faceting *h*_*d*_ and shrinking *h*_*s*_ as a function of the overall pressure ${\rm{\Pi }}$, we see a crossover in the response. At low pressures, *h*_*d*_ is dominant, indicating a response governed by faceting (Fig. 6b–d). As the pressure is increased, we see a transition in the behaviour, as a crossing point of *h*_*s*_ and *h*_*d*_, to a response governed by isotropic shrinkage. This is in qualitative agreement with our experimental observations. These results can seem counterintuitive from the point of view of classical contact mechanics, where the contact between two spheres would always increase its facets if they are more compressed. However, here we are dealing with a particle compressed and surrounded by other particles. As such, both contact mechanics and osmotic effects come into play.

The crossover pressure at which ${h}_{s} > {h}_{d}$ thus signals the point at which the osmotic effects of the particle suspension as a whole begin to dominate over the contact mechanics at the particle-particle contacts. At pressures beyond this crossover point, the high osmotic pressure of the particle ‘bath’ leads to a homogeneous deswelling of the particles.

Within the model we have choosen here, the ratio of shrinkage versus deformation depends strongly on the Poisson ratio of the microgels. For low Poisson’s ratios, indicative of compressible solids, shrinkage begins to dominate at relatively low pressures. By contrast, when we choose a high Poisson’s ratio, close to that for an incompressible solid, a response governed by deformation is observed. In fact, in the limit of $\nu \to 0.5$, *h*_*s*_ goes to zero and $h\approx {h}_{d}$.

Experimentally, we find a crossover in the sphericity at approximately 10 kPa. While the model is approximate, e.g. by the choice of a mean-field approach for the osmotic pressure and ignoring the molecular details of the particle surface, comparing this value to the theoretical cross-over pressures, indicates that the Poisson ratio of our experimental system is between 0.43–0.45. This is in the correct order of magnitude for swollen polyacrylamide hydrogels, for which $\nu =0.457$, as determined independently previously^33^.

At this time, the agreement between theory and experiments is qualitative, since the exact equation of state is not known for these particles. While the Flory-Rehner form (Eqs 2–5) is a common starting point, it does not take microscopic details, such as crosslinking inhomogeneities, the effects of charges, etc. into account. It may be expected that changing the exact nature of the equation of state, or of the expressions used to relate the network structure to the shear rigidity, will change the crossover pressure at which osmotic effects begin to govern over contact mechanics. However, the general notion that at low overal osmotic pressure the particle-particle contacts themself dominate the particle deformation, while the bath pressure takes over when it becomes sufficiently large, is expected to hold irrespective of the choices for the equation of state. In fact, since the facetting is most sensitive to the Young’s modulus of the particles, while homogeneous deswelling is governed by their bulk modulus, we may speculate that the Poisson ratio of the particle is the governing metric for if and when a crossover in behaviour may be expected.

Depending on the manner in which microgels are prepared, the surface structure of the polymer chains may be different, leading to significant variations in the length and grafting density of the dangling surface polymers. Also this can have an effect that is currently not accounted for, e.g. by the creation or suppression of lubrication layers and the establishment of a significant disjoining pressure to break these layers during compression.

Finally, recent work from our group has suggested an approach to treat the real volume fraction in systems of compressible colloids but taking osmotic deswelling into account^18^, where we assumed that only deswelling occurs while facetting was presumed to be negligible. The results in the current study highlight that this approximation fails especially close to the jamming transition where facetting is severe. Interestingly, since facetting does not lead to a reduction in the real particle volume fraction with compression while deswelling does, the crossover in behaviour we find indicates an even steeper effect of compression on the real versus apparent volume fraction than that predicted previously^18^. Moreover, the purpose of the previous study was to explore the effect of osmotic deswelling in absence of facets, on the slowing down of structural relaxations in microgel glasses. We may expect that facets, and the lubrication layers between the two interfaces across a facet, could alter the diffusion rate of particles with respect to their neighbours, and thus have a pronounced effect on the nature of the colloidal glass transition. In principle, this could be tested by comparing the behaviour of particles with identical stiffness but different Poisson ratios, through which the balance between osmotic versus contact effect can be tuned.

## Conclusion

In this paper, we investigated the behaviour of individual microgels in microgel packings under compression considering simultaneous deswelling and deformation mechanisms. Our experiments show that microgels initially facet under compression and that at higher compression pressures, they regain their spherical shape. To explain this behaviour, we propose a model that balances the work of osmotic deswelling, within the Flory-Rehner picture of gel swelling, versus facet formation in the Herzian contact model. Numerical solutions of the model predict behaviour qualitatively consistent with our experimental observations with a crossover from contact mechanics dominated response at low pressures to an osmotically governed response at high pressures. These results imply that treatments of the dynamics and mechanics of packings of soft particles, that account only for facetting or deswelling, are approximate, and that a full description requires taking both effects into account. This is particularly important at low pressures, close to the jamming and/or glass transition, where deformations are significant. These results also have important implications for the flow behaviour of soft particles, e.g. in complex geometries such as membrane pores or constrictions^34–37^, where deswelling and/or deformation plays an important role in pore passage and mitigation of clogs.

## Material and Methods

### Microgel synthesis

We synthesise polyacrylamide microgels by polymerization of monomer solutions in emulsion droplets as a template. In a round bottom flask, we mix 100 ml kerosene with 1%wt of the surfactant polyglycerol polyricinoleate (PGPR90). In a separate flask we prepare our monomer solution with 10 ml of water, 0.1 M sodium hydroxide solution to set the pH at 8.5, 2.5 g of acrylamide, 50 mg of potassium persulfate (KPS) and 25 mg of N,N′-methylenebisacrylamide (BIS) as the crosslinker at 1%wt as compared to the total monomer content. For fluorescent microgels, we include 25 mg of fluorescein methacrylate at this stage. We add our monomer solution to the content of the round bottom flask and emulsify the aqueous phase into the oil phase under high shear with a rotor-stator mixer for three minutes. We then close the round bottom flask with a rubber septum and bubble the emulsion with nitrogen for 20 minutes to remove oxygen. We subsequently place the round bottom flask on a stirring plate on ice and we inject 1 ml N,N,N′,N′-tetramethylethylenediamine (TEMED) to trigger the polymerization. We allow the system to react for 2–3 hours and precipitate the microgels in cold methanol. We clean our microgels by repeated centrifugation and resuspension steps, first in methanol to remove excess kerosene and surfactant, and finally in water, after which the microgel suspension is stored at 4 °C.

### Osmotic stress

We use a mixture of fluorescent and non-fluorescent microgel suspensions at a number ratio of 1:20 to allow observation of individual microgels in the packing using confocal fluorescence microscopy. We compress the microgel suspension using the osmotic stress technique. We place the suspension of microgels in dialysis bags that we then place in a solution of polyethylene glycol (PEG) with known concentration. The concentration of a PEG solution can be correlated to its osmotic pressure through empirical equations available in the literature^38^. We use a range of PEG concentrations corresponding to osmotic pressures between 2.5 kPa and 2 MPa. The volume of dialysate is at least 100 times larger than the sample volume. The system is allowed to equilibrate for two weeks to ensure the desired compression pressure *P* is achieved. The dialysate is renewed in the middle of this process, after one week.

### Confocal microscopy

To determine how the microgels behave we use confocal fluorescence microscopy to record three-dimensional image stacks of individual, fluorescently labelled microgels. As we only have a small amount of fluorescently labelled microgels in each sample we can visualize single microgels as they deform and shrink at varying osmotic pressure. These experiments are performed on a Zeiss microscope, equipped with a 488 nm laser line and imaged using a × 100 oil-immersion objective. The resolution of the images is 1000 × 1000 pixels. To measure the type and degree of deformation of a microgel at different compression pressures we analyse the confocal image stacks using custom Matlab routines (available upon request). To accurately determine the surface area and circumference of a microgel in each confocal slice we trace the boundary of every microgel and fit a polynomial function to this shape using Savitsky-Goley smoothing. We calculate the surface area for each slice in our three-dimensional image stack and determine the total volume of each microgel in our field of view.

## References

[1] Fernández-Nieves A, Fernández-Barbero A, Vincent B, De Las Nieves FJ (2000). Charge controlled swelling of microgel particles. Macromolecules.

[2] Han K, Tiwari R, Heuser T, Walther A (2016). Simple Platform Method for the Synthesis of Densely Functionalized Microgels by Modification of Active Ester Latex Particles. Macromolecular Rapid Communications.

[3] Destribats M (2011). Soft microgels as Pickering emulsion stabilisers: role of particle deformability. Soft Matter.

[4] Sung B, Kim C, Kim MH (2015). Biodegradable colloidal microgels with tunable thermosensitive volume phase transitions for controllable drug delivery. Journal of Colloid and Interface Science.

[5] Guo M, Wyss HM (2011). Micromechanics of Soft Particles. Macromolecular Materials and Engineering.

[6] Sierra-Martin B (2011). Determination of the bulk modulus of microgel particles. Colloid and Polymer Science.

[7] Mattsson J (2009). Soft colloids make strong glasses. Nature.

[8] Meeker SP, Bonnecaze RT, Cloitre M (2004). Slip and flow in soft particle pastes. Physical Review Letters.

[9] Di Lorenzo F, Seiffert S (2013). Particulate and continuum mechanics of microgel pastes: Effect and non-effect of compositional heterogeneity. Colloid and Polymer Science.

[10] Habicht A (2015). Critical fluctuations and static inhomogeneities in polymer gel volume phase transitions. Journal of Polymer Science, Part B: Polymer Physics.

[11] Nolan CM, Reyes CD, Debord JD, García AJ, Lyon LA (2005). Phase transition behavior, protein adsorption, and cell adhesion resistance of poly(ethylene glycol) cross-linked microgel particles. Biomacromolecules.

[12] Su W, Yang M, Zhao K, Ngai T (2016). Influence of Charged Groups on the Structure of Microgel and Volume Phase Transition by Dielectric Analysis. Macromolecules.

[13] Habicht A, Schmolke W, Lange F, Saalwächter K, Seiffert S (2014). The non-effect of polymer-network inhomogeneities in microgel volume phase transitions: Support for the mean-field perspective. Macromolecular Chemistry and Physics.

[14] Nyström L (2016). Electrostatic Swelling Transitions in Surface-Bound Microgels. ACS Applied Materials and Interfaces.

[15] Maldonado-Valderrama J, del Castillo-Santaella T, Adroher-Benítez I, Moncho-Jordá A, Martín-Molina A (2016). Thermoresponsive microgels at the air–water interface: the impact of the swelling state on interfacial conformation. Soft Matter.

[16] Pellet C, Cloitre M (2016). The glass and jamming transitions of soft polyelectrolyte microgel suspensions. Soft Matter.

[17] Lietor-Santos JJ (2009). Deswelling microgel particles using hydrostatic pressure. Macromolecules.

[18] van der Scheer P, van de Laar T, van der Gucht J, Vlassopoulos D, Sprakel J (2017). Fragility and Strength in Nanoparticle Glasses. ACS Nano.

[19] Mohanty PS (2017). Interpenetration of polymeric microgels at ultrahigh densities. Scientific Reports.

[20] Hashmi SM, Dufresne ER (2009). Mechanical properties of individual microgel particles through the deswelling transition. Soft Matter.

[21] Savitzky A, Golay MJE (1964). Smoothing and Differentiation of Data by Simplified Least Squares Procedures. Analytical Chemistry.

[22] Liétor-Santos JJ, Sierra-Martín B, Fernández-Nieves A (2011). Bulk and shear moduli of compressed microgel suspensions. Physical Review E - Statistical, Nonlinear, and Soft Matter Physics.

[23] de Gennes, P. *Scaling Concepts in Polymer Physics* (Cornell University Press, 1979).

[24] Flory, P. *Principles of Polymer Chemistry*. Baker lectures 1948 (Cornell University Press, 1953).

[25] Menut P, Seiffert S, Sprakel J, Weitz DA (2012). Does size matter? Elasticity of compressed suspensions of colloidal- and granular-scale microgels. Soft Matter.

[26] Scotti A (2016). The role of ions in the self-healing behavior of soft particle suspensions. Proceedings of the National Academy of Sciences.

[27] Flory PJ, Rehner J (1943). Statistical Mechanics of Cross-linked Polymer Networks I. Rubberlike Elasticity. The Journal of Chemical Physics.

[28] Flory PJ, Rehner J (1943). Statistical Mechanics of Cross-linked Polymer Networks II. Swelling. The Journal of Chemical Physics.

[29] Seth JR, Mohan L, Locatelli-Champagne C, Cloitre M, Bonnecaze RT (2011). A micromechanical model to predict the flow of soft particle glasses. Nature Materials.

[30] Kundu S, Crosby AJ (2009). Cavitation and fracture behavior of polyacrylamide hydrogels. Soft Matter.

[31] Kayaman N, Okay O, Baysal BM (1998). Swelling of polyacrylamide gels in polyacrylamide solutions. Journal of Polymer Science Part B: Polymer Physics.

[32] van Hecke M (2010). Jamming of soft particles: geometry, mechanics, scaling and isostaticity. Journal of Physics: Condensed Matter.

[33] Takigawa T, Morino Y, Urayama K, Masuda T (1996). Osmotic Poisson’s Ratio and Equilibrium Stress of Poly(acrylamide) Gels. Polymer Journal.

[34] Linkhorst, J., Beckmann, T., Go, D., Kuehne, A. & Wessling, M. Microfluidic colloid filtration. *Scientific Reports* **6** (2016). 10.1038/srep22376 PMC4772133 26927706

[35] Hendrickson, G. & Andrew Lyon, L. Microgel translocation through pores under confinement. *Angewandte Chemie - International Edition* **49**, 2193–2197 Cited By 38 (2010). 10.1002/anie.200906606 PMC3056332 20183836

[36] Nir O, Trieu T, Bannwarth S, Wessling M (2016). Microfiltration of deformable microgels. Soft Matter.

[37] Roa R, Zholkovskiy EK, Nagele G (2015). Ultrafiltration modeling of non-ionic microgels. Soft Matter.

[38] Bouchoux A, Cayemitte P-E, Jardin J, Gésan-Guiziou G, Cabane B (2009). Casein Micelle Dispersions under Osmotic Stress. Biophysical Journal.

